# Single-Cell RNA Sequencing Transcriptomics Revealed HCMV IE2-Related Microglia Responses in Alzheimer’s-Like Disease in Transgenic Mice

**DOI:** 10.1007/s12035-023-03553-y

**Published:** 2023-09-13

**Authors:** Fengjun Liu, Zhifei Wang, Delei Niu, Xianjuan Zhang, Fulong Nan, Shasha Jiang, Jun Li, Meng Yu, Xiaoli Yang, Shuyun Zhang, Xiaoqiong Zhou, Hui Wang, Xueming Zhang, Wenxuan Liu, Zonghui Li, Yunyang Wang, Bin Wang

**Affiliations:** 1 Department of Special Medicine, School of Basic Medicine, Qingdao University, Qingdao, 266000 China; 2 Clinical Laboratory, Qingdao Women and Children’s Hospital of Qingdao University, Shandong, Qingdao, 266000 China; 3 Department of Pathogenic Biology, School of Basic Medicine, Qingdao University, Qingdao, 266000 China; 4 Department of Endocrinology and Metabolism, the Affiliated Hospital of Qingdao University, Shandong, Qingdao, 266000 China; 5 Department of Clinical Laboratory, The Affiliated Hospital of Qingdao University, Qingdao, Shandong China

**Keywords:** Human cytomegalovirus (HCMV), Immediate-early 2 (IE2) protein, Alzheimer’s disease, Microglia, Transgenic mice

## Abstract

Although multiple factors are known to concur with Alzheimer’s disease (AD), the relationship between human cytomegalovirus (HCMV) and AD-like disease is unclear. Here, we propose a hypothesis that HCMV immediate-early 2 (IE2) protein promotes microglia activation and thus leads to AD-like disease. We successfully constructed IE2 transgenic mice expressing IE2 in the hippocampus. Single-cell sequencing analysis revealed that IE2 promoted the activation of microglia and upregulated the expression of disease-associated microglia genes. Differentially expressed gene analysis and pathway enrichment revealed that IE2 upregulated immune and nervous system disease-related genes. Immunohistochemical analysis showed that the expressions of both amyloid precursor protein (APP) and p-Tau were significantly upregulated in the brains of IE2 mice and were markers of AD. Taken together, these findings provide useful insights into AD-like disease activated by HCMV IE2.

**Supplementary Information:**

The online version contains supplementary material available at 10.1007/s12035-023-03553-y.

## Introduction 

Alzheimer’s disease (AD) is a neurodegenerative disease with slow progression, which begins with mild memory loss and eventually severely impairs executive and cognitive functions [1, 2]. AD often manifests histologically by the parenchymal deposition of amyloid-beta (Aβ) plaques, the formation of neurofibrillary tangles (NFT) and the presence of neuroinflammation [1, 3]. Several factors may increase the chances of developing AD and, among the “environmental” risk factors, persistent brain infection caused by herpes viruses seemed to play a key role in AD pathogenesis [4].

Human cytomegalovirus (HCMV), belonging to the β-herpesvirus subfamily, infected more than 90% of people in China[5]. HCMV immediate-early 2 (IE2) protein is one of the first protein to be expressed after virus infection and is believed to control all subsequent early and late events in HCMV infection [6, 7]. Previous studies have shown that IE2 impaired the self-renewal and proliferation of neural progenitor cells in vitro [8]. The expression of IE2 in the mouse hippocampus affects the development of the brain, resulting in the thinning of the cerebral cortex and neuroinflammation in mice [9] and causes learning and cognitive impairment in older mice [9, 10]. However, the reason is still unclear between IE2 and cognitive impairment.

Microglia are specialist immune sentinel cells in the brain that respond to stranger or danger signals, remove cellular and extracellular debris, and regulate synaptic plasticity, maturation, and removal [11–15]. They are the main source of cytokines in AD, which contribute to the development of neuroinflammation [16]. Local neuroinflammation associated with cytotoxicity and disease progression in AD is generally attributed to microglia [17]. Single-cell analysis can further identify potential markers, pathways and regulatory factors, and promote testable hypotheses. With the development of single-cell sequencing, growing evidence now points to glial responses to pathology in the AD brain [18]. Therefore, the function of microglia is crucial to the physiological processes in the brain.

Here, an IE2 transgenic mouse animal model successfully constructed in our laboratory [9]. Based on this model, single-cell RNA sequencing was used to examine the heterogeneity of hippocampal cells in the healthy brain and in the brain of HCMV IE2 transgenic mice. In IE2 transgenic mice, we find that microglia activation is a response to IE2 expression and identified a disease associated microglia (DAM) subtypes. Our results also depict the pathways activated in DAM, which have been associated with known AD risk factors and further confirmed by immunohistochemistry. Overall, our study identifies a potential microglia type associated with IE2, which can cause mouse neurodegeneration and provides a new idea for the occurrence and development of AD-like disease by virus infection.

## Materials and Methods

### Animals

HCMV IE2 transgenic mice (on a C57/BL6 background) that overexpressed HCMV IE2 in the hippocampus were constructed and purchased from Shanghai Model Organisms (Shanghai, China). Six-month-old and 12-month-old mice (on a C57/BL6 background) were purchased from SPF (Beijing) Biotechnology Co., Ltd and used for lentivirus infection. Mice were bred and maintained by the Animal Breeding Center of the Qingdao University. All experiments detailed herein complied with the regulations formulated by the Institutional Animal Care and Use Committee of Qingdao University.

### Construction of Lentiviral Vectors

Lentivirus-HCMV IE2 (LV-IE2) vectors were constructed according to the manufacturer’s protocol (GeneChem Inc.) and purchased from GeneChem Co., Ltd (Shanghai, China). The sequence of LV-IE2 is listed in Table 1. The titer of the lentiviral vectors was 2.5 × 10^8^ TU/mL. Lentivirus vectors coding for GFP were used as control vectors.

**Table 1:** The sequences of LV-IE2

Name	Sequences
LV-IE2-P1	AGGTCGACTCTAGAGGATCCCGCCACCATGGAGTCCTCTGCCAAGAG
LV-IE2-P2	ACCGTAAGTTATGTGCTAGCTTACTGAGACTTGTTCCTCAGGTC

### Intrahippocampal Injection of Lentiviral Vectors

For brain injection of the virus, 6-month-old and 12-month-old C57 mice (*n* = 6) were anesthetized with isoflurane via intraperitoneal injection and then positioned in a stereotaxic instrument. LV-IE2 or LV-GFP (2 µL) were injected into the hippocampus (ML 1.5, AP 2.0, DV − 2.0) at a rate of 0.2 µL/min. The syringe was left in place for 3 min before being slowly withdrawn from the brain. Two weeks after the intrahippocampal injection, one mouse was randomly selected from each experimental group and analyzed for GFP expression to verify successful lentivirus infection in the hippocampus.

### Tissue Harvesting and 10 × Genomics Chromium Single-Cell 3’ Library Construction

Mice were transcardially perfused with PBS before hippocampal extraction (*n* = 3). The single-cell 3’ library was constructed according to the instructions given in the Chromium Next GEM Single-Cell 3’ Reagent Kit (v.3.1, BerryGenomis, China). All procedures were carried out on ice or at 4 °C. To achieve single-cell resolution, cells were delivered at a limiting dilution, such that the majority of the generated gel beads in emulsion (GEMs) contain no cells, while the remainder largely contained a single cell. Immediately following GEM generation, the gel bead was dissolved, and the primers (Illumina TruSeq Read 1, 16nt 10 × Barcode, 12nt unique molecular identifier, 30nt poly(dT) sequence) were released, while any co-partitioned cells were lysed. Incubation of the GEMs produces barcoded, full-length cDNA from poly-adenylated mRNA. After incubation, GEMs were broken, and pooled fractions were recovered. Silane magnetic beads were used to purify the first-strand cDNA from the post GEM-RT reaction mixture, which included leftover biochemical reagents and primers. Barcoded, full-length cDNA was amplified via PCR to generate a sufficient mass for library construction. Enzymatic fragmentation and size selection were used to optimize the cDNA amplicon size. P5, P7, i7, and i5 sample indexes, as well as TruSeq Read 2 (Read 2 primer sequence), were added via end repair, A-tailing, adaptor ligation, and PCR. The final libraries contain the P5 and P7 primers, which were used in the Illumina amplification. The chromium single-cell 3’ gene expression dual index library comprised standard Illumina paired-end constructs, which began and ended with P5 and P7. The 16 bp 10 × Barcode and 12 bp UMI were encoded in Read 1, while Read 2 was used to sequence the cDNA fragment. The index sequences, i7 and i5, were incorporated as the sample index reads. TruSeq Read 1 and TruSeq Read 2 are standard Illumina sequencing primer sites used in paired-end sequencing.

### Data Analysis and Visualization

Sequence data were analyzed by Cell Ranger or 10 × Genomics Cloud Analysis and visualized by Loupe Browser. Barcoded reads were demultiplexed and aligned to the Mouse (MM10) genome with Ensemble transcriptome annotation using CellRanger with default parameters. We determined 200UMIs as the lower cut-off for cell filtration and kept for clustering and downstream analysis.

### Cell Clustering

All 14,500 cells were combined into a single dataset. Normalization and clustering were done with the SCANYP package [19]. Putative cell types were identified using an iterative clustering approach. Principal component analysis (PCA) was performed on the variable genes, t-SEN was run on the top 10 principal components (PCs) using the Multicore-TSEN package.

### Cell Type Annotation

To identify the major cell types, the FindAllMarkers function was used to enrich DEGs in one cluster compared to all other clusters. These cluster-specific genes were then queried against a set of canonical cell type-specific markers from the literature. Data were visualized using Loupe Browser 6.

### Functional Annotation of the Different Microglia Sub-clustering

For sub-clusters, a set of markers (specifically overexpressed) genes were defined by a differential expression analysis of the cells grouped in each sub-cluster against the remaining cells within the corresponding broad cell-type cluster.

The top 5 genes of homeostatic microglia and disease-associated microglia were submitted to downstream analysis. Sub-clusters were defined by grouping together all pre-clusters corresponding to the same cell type.

### Cluster-Specific Differential Gene Expression and Pathway Analysis

DEG analysis was completed by edgeR package, including calculation of normalized factors, estimation of dispersion, and model fitting using glmFit [20], comparing disease state in microglia (WT versus IE2), and passing zinbwave-generated observational weights to the glmWeightedF function. Genes were identified as DEGs if they had an adjusted *p*-value < 0.05 using the Benjamini–Hochberg method and had a log2 fold change > $\pm$ 0.25 [21].

For pathway analysis, all DEGs were converted to their Ensembl IDs. Subsequently, their Entrez IDs prior to being separated into upregulated and downregulated lists with their accompanying log2 fold changes. Each list was then analyzed separately to determine upregulated and downregulated GO term and KEGG pathway. Upregulated and downregulated DEGs, GO terms as well as KEGG pathways were compared among clusters and were visualized using *UpSetR* package. Lists of DEGs and pathways were provided in Supplement Table.

### Pseudotime Analysis

To obtain pseudo-temporal ordering of the cells along the transition from homeostatic microglia to DAM. The Monocle package (version 2) was used for pseudotiming analysis. We selected representative homeostatic microglial cells (from WT mice) and representative DAM cells (from IE2 mice) as waypoints. For each cell, we calculated the shortest path to each of the waypoints and sorted the cells according to their distance. The final trajectory was an average over all graph trajectories [17].

### Immunohistochemistry

The protocol of immunohistochemistry was performed with minor adjustments as previously described [22]. Mice were transcardially perfused with PBS and 4% paraformaldehyde (PFA) before brain extraction. Mouse brain paraffin embedded sections were 10-µm thick. The following primary antibodies were used: rabbit anti-APP, rabbit anti-p-Tau-T205, rabbit anti-GFP (Abclonal, China) at 1:50. A goat anti-rabbit IgG secondary antibody were used for detection.

### Immunofluorescence

Brain tissues were fixed with 4% PFA and serial 10-µm-thick paraffin-embedded sections were obtained. Immunofluorescence staining was performed as detailed previously [9]. The following primary antibodies were used: rabbit anti-IBA-1, mouse anti-CD11C, mouse anti-CSF1, mouse anti-CX3CR1 (Abconal, China). The secondary antibodies used were Goat anti-Mouse IgG (H + L) Alexa Fluor 488 (Invitrogen, USA), Goat anti-Mouse IgG (H + L) Alexa Fluor 555 (Invitrogen, USA), Goat anti-Rabbit IgG (H + L) Alexa Fluor 488 (Invitrogen, USA), and Goat anti-Rabbit IgG (H + L) Alexa Fluor 555 (Invitrogen, USA).

### Statistical Analysis

The single-cell RNA sequencing data for two groups were calculated using Student’s *t*-test. *P* values below 0.05 were considered significant. The heatmaps were performed using GraphPad Prism V9.0 (GraphPad Software, Inc, La Jolla, CA).

## Results

### Nature and Distribution of the Single-Cell RNA Sequencing Data

**Fig. 1:**
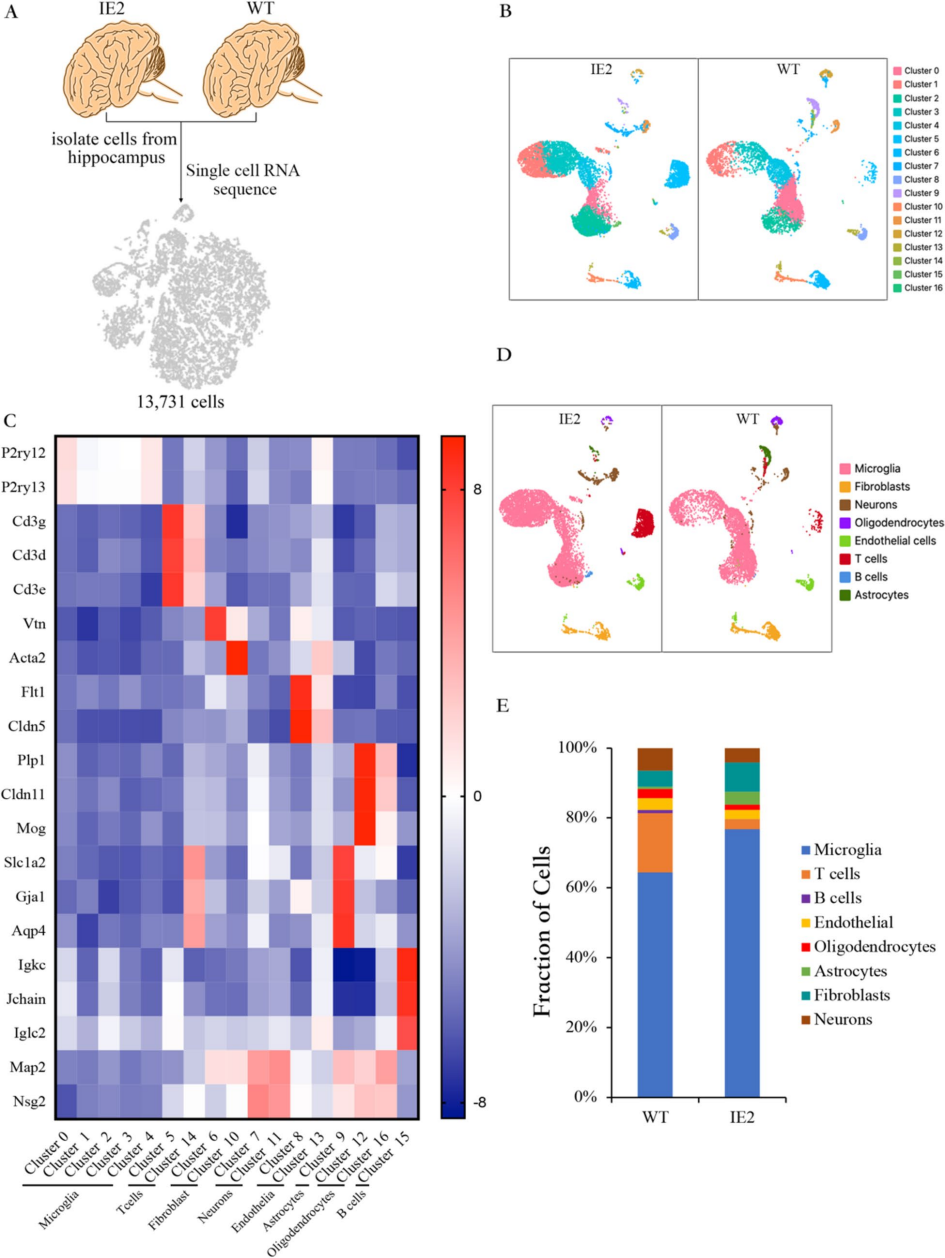
Single-cell RNA sequencing of hippocampus in IE2 transgenic mice and WT mice. **A** Single cell RNA sequencing workflow of cells isolated from the hippocampus of IE2 transgenic mice and control littermates. **B** Cell map of mouse hippocampus. UMAP plot showing 16 clusters from 3 IE2 transgenic mice and 3 WT mice. Each dot represents a cell. *n* = 13,731 total cells. **C** Heat map showing expression of specific markers in every cluster, identifying each cluster in B. **D** UMAP plot depicting the different microglial and non-microglial cell clusters. Each dot represents a cell. The cells are color coded based on their cluster affiliation. **E** Bar graph showing the frequency of each cluster in IE2 and WT mice

To investigate the phenotypic heterogeneity and the transcriptional dynamics of hippocampal during the progression of neurodegeneration caused by HCMV IE2, single-cell RNA sequencing was conducted to analyze the differences between IE2 transgenic mice and wide-type (WT) mice. We isolated cells from hippocampi of three IE2 transgenic mice and three C57/BL6 mice (control). A rigorous pre-processing pipeline (see “Materials and Methods” section) yielded transcriptomes from a total of 14,585 individual cells, including 8238 from IE2 transgenic mice and 6347 from C57/BL6 mice (Fig. 1A). We performed robust batch correction on our data using standard regression models. Subsequently, we performed the iterative PCA Louvain clustering method using a stepwise clustering robustness assessment and identified 17 distinct cell clusters, with a minimum of 44 cells per cluster. Our analysis identified a large group of glial cells (microglia in clusters 0–4, astrocytes in cluster 9, oligodendrocytes in clusters 12 and 16), neurons (clusters 7 and 11), several lymphocyte subgroups (B cells in cluster 15, T cells in clusters 5 and 14), and other cell types (fibroblasts in clusters 6 and 10, endothelial cells in clusters 8 and 13) (Fig. 1B–C).

Examining the contribution of WT versus IE2 background to each group of cells revealed a large difference in the percentage of cells in microglia and T cells. The numbers of microglia and T cells in the IE2 groups were significantly higher compared to those of the WT group (Fig. 1E). Visualization of the cells using the t-SEN map further supported the separation of microglia and non-microglia clusters (Fig. 1D). Because genetic studies have emphasized the important role of microglia in the susceptibility to different neurodegenerative diseases [23, 24], we paid special attention to the changes in microglia in the IE2 transgenic mice.

### The DAM Signature is IE2 Dependent

**Fig. 2:**
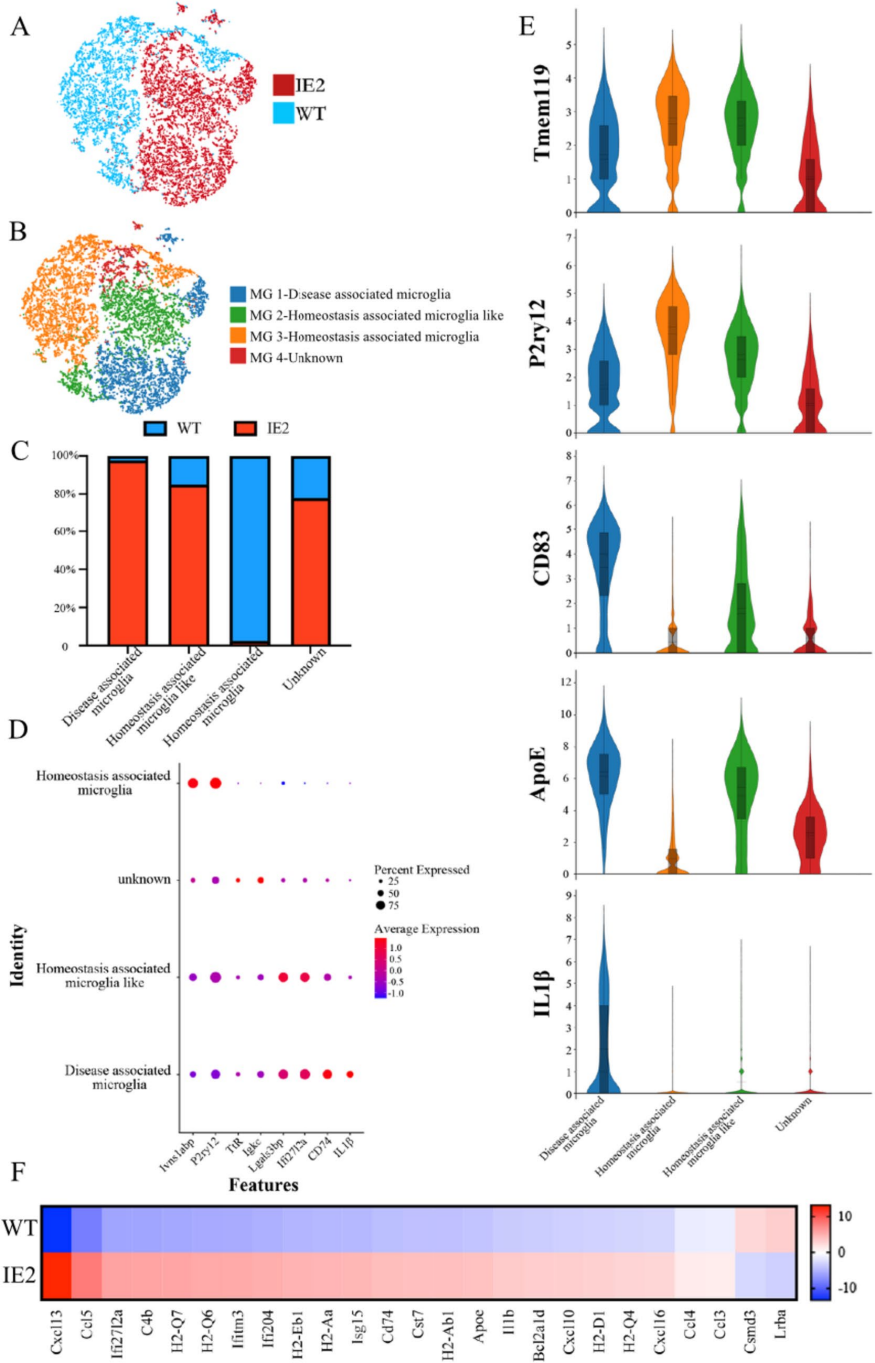
Functional annotation of the microglial clusters. **A** t-SEN plot showing the microglia cluster (clusters 0–4) from Fig. 1 expressing microglia genes, such as P2ry12 and P2ry13. Each dot represents a cell, red dots represent cells derived from IE2 transgenic mice and blue dots represent cells derived from WT mice. *n* = 10,290. **B** t-SEN plots of reanalyzed microglia from published snRNA-seq datasets. Microglia are annotated as 4 sub-cluster. Each dot represents a cell, bule dots represent disease associated microglia, green dots represent homeostasis associated microglia-like, orange dots represent homeostasis associated microglia, and red dots represent unknown cells. **C** Bar graph showing the frequency of each microglia subcluster in every sample. Red represents cells derived from IE2 transgenic mice and blue represents cells derived from WT mice. **D** Dot plot of canonical genes to classify tSNE clusters MG1-4 (from Fig. 2B). Cluster identities are labeled on the left and canonical marker genes are located on the bottom. Darker shades of red and size of dots represent greater expression of genes and percentage of cells expressing the gene. **E** Violin plots showing the expression of homeostatic and DAM genes. CD83, ApoE, and IL1ß are mainly enriched in disease associated microglia (MG 1). Violin plots are presented with floating boxes showing median (middle line) and quartiles (top and bottom). **E** Heat map showing expression of specific markers in microglia in IE2 and WT mice

**Fig. 3:**
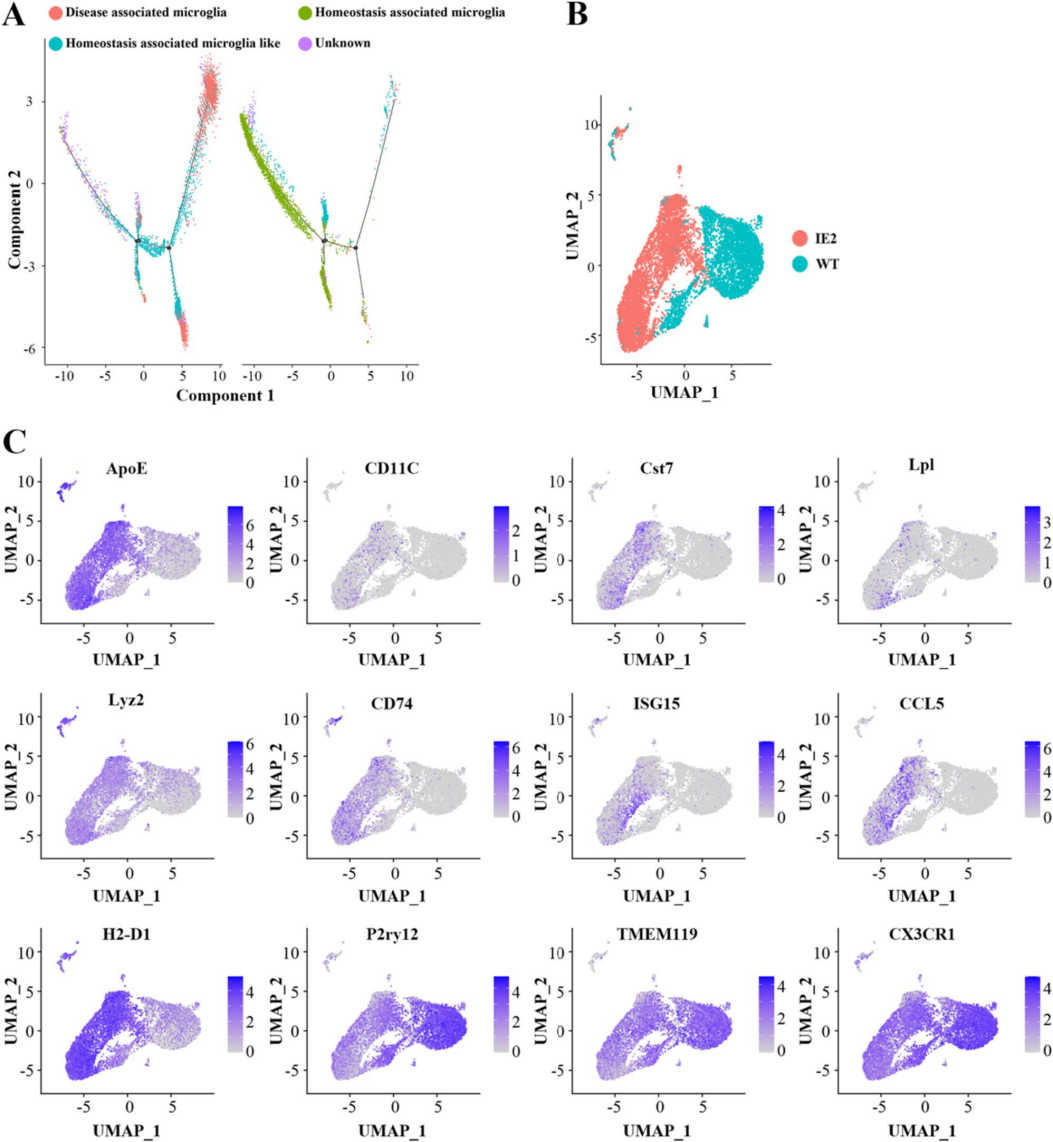
Disease associated microglia display dynamics of activation during IE2 expression. **A** Continuous locus of three major microglial states in IE2 and WT brains. 10,290 microglia were embedded in the diffusion map (as shown in Fig. 2A), highlighting the cells under IE2 and WT conditions, and colored according the inferred cluster identity. Left: Schematic diagram of the state and predicted transformation of microglia in WT. Right: Schematic diagram of the state and predicted transformation of microglia in IE2. **B** UMAP embedding microglia in hippocampus of WT (left) and IE2 (right). **C** Projection of key marker genes onto the graph plot of microglia, as shown in **B**. Color bar below each plot represents color scale level (log_2_ UMI counts)

Next, we studied the microglia subtypes. According to specific cell markers and projection of the cells using t-SEN, microglia were divided into four subclusters (Fig. 2A–B). Annotation analysis of the microglia subclusters showed that the number of microglia was significantly increased in MG1 and MG2, and markedly decreased in MG3, compared with the WT (Fig. 2C). A closer examination of the maps and key marker genes of the microglia in the subclusters revealed that there were significant differences between the gene expression in the microglia (Fig. S1). Cluster-enriched sets of transcription factors and transcriptional regulators were found in MG1 and MG2. Many more microglia activation-related genes were upregulated in MG1 and MG2, including known AD risk factors (ApoE) [25] and immune response-related genes (CD74, H2-Aa and H2-Ab1) [26] (Fig. 3D–E). The lack of detectable distinct on-off transcription factors and cell surface markers in MG3 was consistent with our hypothesis that this cluster may represent homeostatic microglia and were mainly present in WT mice. Two-dimensional projection of the graph identified homeostatic microglia and IE2-related microglia on the two extremes of the graph, with an intermediate group of cells (MG2-homeostasis associated microglia-like) connecting the two states.

Next, we turned to the annotation of the microglia clusters using other signatures reported in the literature. 5 × FAD mice have been widely in the study of AD, and a recent study of the 5 × FAD mouse model demonstrated the activation of microglia in diseased brain [17]. These activated microglia are also known as DAM and have different transcriptional signatures compared to homeostatic microglia [26]. Our results showed that DAM genes, including ApoE, Lpl, Cst7, Csf1, H2-d1, Cd74, and various cathepsin genes, were notably upregulated in IE2 transgenic mice compared with WT mice. These results were highly consistent with the previously published single-cell RNA-seq data of sorted microglia [16, 17, 27]. We also identified interferon response genes (Ifitm3, IRF7) and chemokines (CCL3, CCL4, CXCL16) (Fig. 2F). This analysis demonstrated that microglia in the IE2 model display progression to a disease state. Thus, we proposed a model of microglia population structure and highlighted one subtype whose frequency is altered in IE2-expressed brain.

### Disease-Associated Microglia Dynamics During IE2 Expression

Neurodegenerative disease is progressive, with neuron death and cognitive loss increasing gradually [28]. Determining the relevant changes in DAM regulation of IE2 in the course of disease can clarify the molecular mechanism of IE2 in the regulation of neurodegenerative disease and may suggest new diagnostic and therapeutic goals. In order to simulate the development of microglia in the progression of disease, we performed a pseudo time analysis of microglia in IE2 and WT mice (Fig. 3A). Through joint analysis of the control groups and the experimental groups, it was found that, with the expression of IE2, the state of microglia gradually changed from the homeostatic state to an intermediate state and finally to the diseased state (Fig. 3B). Comparison of homeostatic-associated microglia with DAM showed that the expression changes for many of the DAM-specific genes were in the same trajectory but were more pronounced in the DAM cluster, which may suggest that the homeostatic-associated microglia-like cluster is an intermediate state between homeostatic-associated microglia and DAM.

Further analysis of microglia in the three states showed that microglia in the IE2 model, but not in WT, showed the transition from homeostatic microglia to DAM. While most genes did not change their expression, some genes displayed a decrease in expression along this activation axis (P2ry12, CX3CR1), while others show increased expression (ApoE, CD11C, Cst7, Lpl, Lyz2, CD74, ISG15, CCL15, H2-D1). Combined with the above results, our mouse model demonstrated that the transition of microglia from homeostasis to DAM is IE2-dependent (Fig. 3C).

### Microglia Respond to IE2-Related Neurodegeneration

**Fig. 4:**
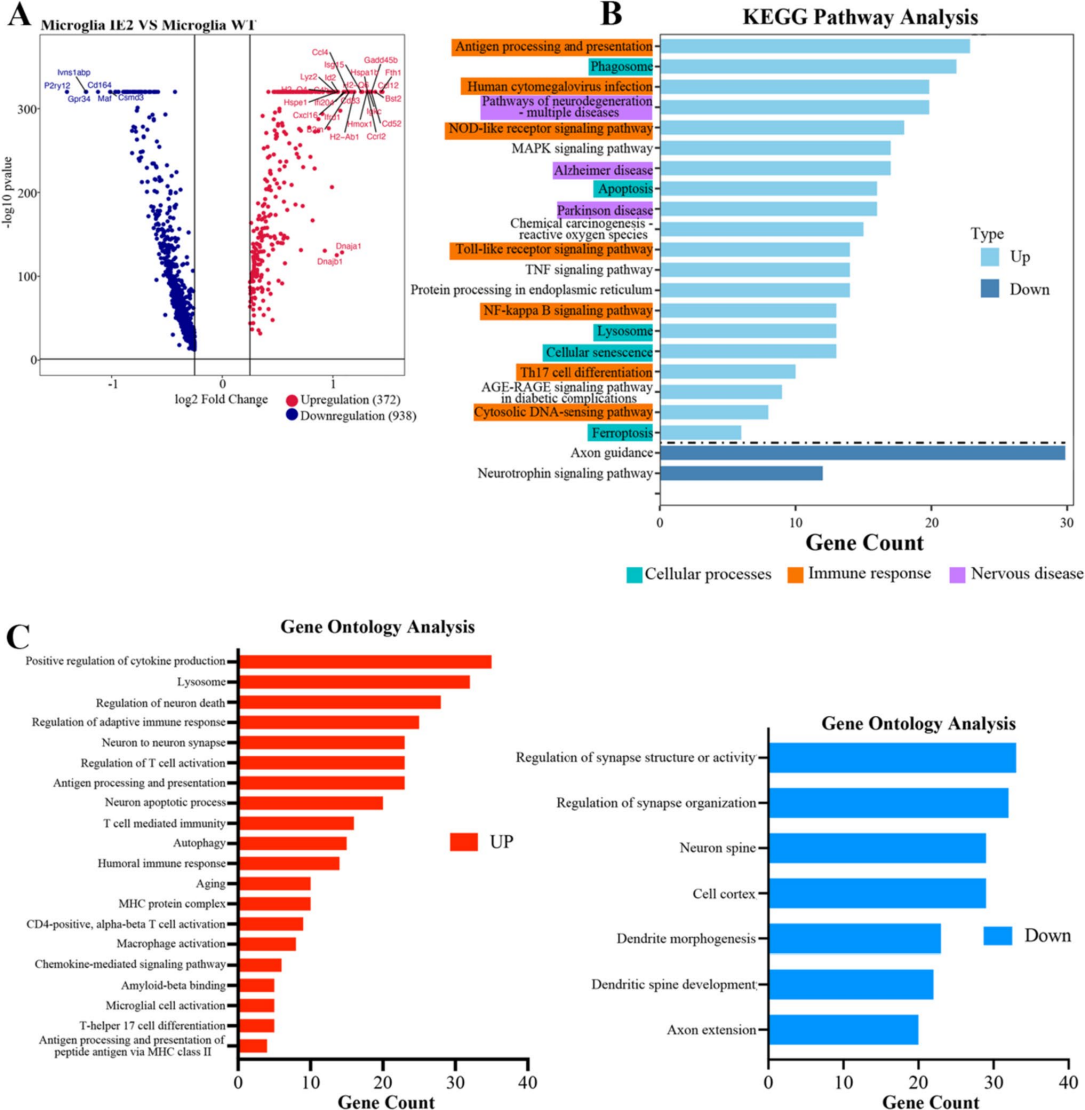
IE2 in microglia is related to cellular stress and immune response pathway. **A** Comparison of log *p*-values of microglia differential expression of IE2 mice versus WT mice. log p-values sign corresponds to upregulation or downregulation. **B** Histogram of KEGG pathways enrichment in microglia of IE2 mice versus WT. Up: Upregulation pathway enriched in KEGG pathway analysis. Down: Downregulation pathway enriched in KEGG pathway analysis. **C** Histogram of GO pathways enrichment in microglia of IE2 mice versus WT. Left: Upregulation pathway enriched in GO analysis. Right: Downregulation pathway enriched in GO analysis

We noticed that IE2 had a great effect on microglia. To further examine the implications of IE2 in microglia, we collected the DEGs related to IE2. We next examined how microglia respond in the IE2-overexpressed brain environment. We detected 372 significantly upregulation and 938 downregulated genes in the microglia of transgenic mice (Fig. 4A). Gene Ontology (GO) term enrichment and KEGG pathway analysis revealed that the IE2 brain upregulated the expression of genes related to immune, neurodegenerative disease, phagosome, and autophagy pathways and downregulated genes related to synaptic trophic development pathway (Fig. 4B–C). While examining the relationships among the IE2-correlated pathways, we found that some pathways were tightly connected into modules and were related to the expression of IE2. For example, one module contained closely interconnected pathways related to neurodegenerative disease, including AD and Parkinson’s disease (Fig. 4B, purple). Additionally, we found a module related to cellular processes, including phagosomes, apoptosis, lysosomes, cellular senescence, and ferroptosis (Fig. 4B, blue). However, the largest module of IE2-related pathways was related to immune response pathways (Fig. 4B, orange). Some of these immune response pathways were related to autoimmunity and infection. These data indicated that IE2 expression is related to the pathways of cellular stress and immune response

### IE2 Upregulated the Expression of AD-Associated Markers

**Fig. 5:**
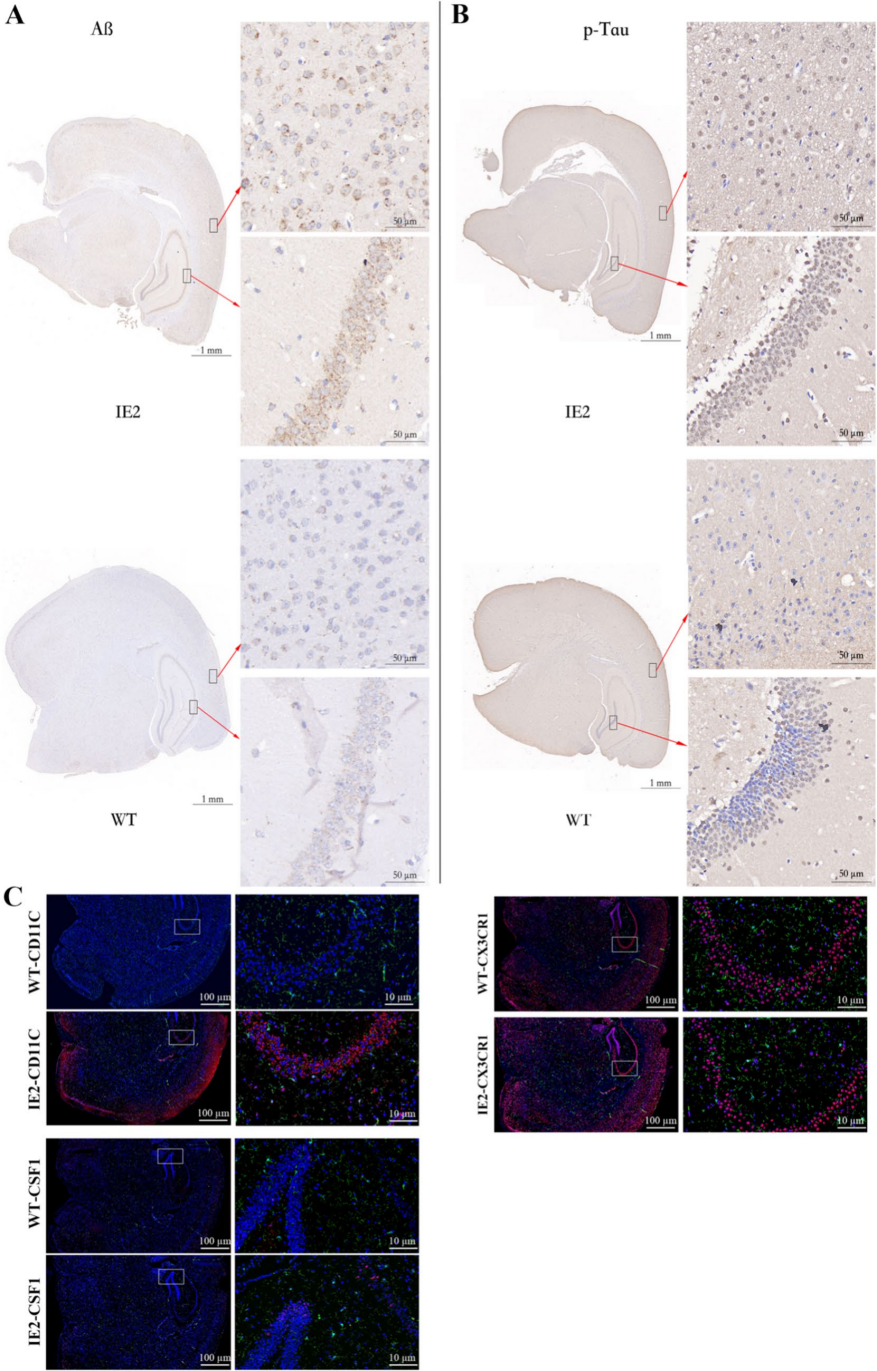
IE2 induces microglial activation and promotes the development of AD-like disease in IE2 transgenic mice. **A** Representative IHC from IE2 mice and age-matched WT controls showing that Aß plaques were upregulated in IE2 mice. The inset shows the 40 $\times$ magnification of the corresponding panel. Scale bar, 1 mm (panels); 50 µm (insets). **B** Representative IHC from IE2 mice and age-matched WT controls showing that p-Tau were upregulated in IE2 mice. The inset shows the 40 $\times$ magnification of the corresponding panel. Scale bar, 1 mm (panels); 50 µm (insets). **C** Microglia expressing DAM markers exist in IE2 brain. Typical immunofluorescence images of classical hippocampal slices of 12-month-0ld LV-IE2 infected mice. The microglial marker IBA-1 of WT mice and IE2 mice was stained green, CD11C, CSF1, and CX3CR1 were stained red, and the nucleus was stained blue. Scale bar, 100 µm (panels) and 10 µm (insets)

According to the comparison between human age and mouse age, 6-month-old mice are equivalent to young or middle-aged humans. However, IE2 can accelerate cerebral neuropathy, so we assumed that IE2 mice had senile nervous system disease. The single-cell sequencing data showed that HCMV IE2 promotes the transition of microglia from homeostasis to diseased state and causes neurodegeneration. We also performed immunohistochemical staining for Aβ plaques and p-Tau in IE2 transgenic mice and WT mice. Aβ plaque formation and the expression of p-Tau were found to be markedly increased in IE2 transgenic mice compared with WT mice in all groups (Fig. 5A–B). C57/BL6 mice were infected with lentivirus (LV)-IE2 and LV-GFP. A month later, mouse brain samples were collected and detected. Aβ plaque formation and the expression of p-Tau had the same expression trend as IE2 transgenic mice (Fig. S2). To further verify our findings, we focused on the differentially expressed DAM markers in IE2 transgenic mice and WT mice. Previous studies have shown that these genes were also upregulated in AD model mice [17]. Hippocampus were stained for IBA-1, a classical homeostatic microglia marker, DAM markers (CD11C and Csf1), and the homeostatic microglia marker (CX3CR1). The expression of CD11C and Csf1 were found to be significantly increased in IE2 transgenic mice compared with WT mice, while CX3CR1 expression was slightly decreased in IE2 mice (Fig. 5C).

### The Effect of IE2 on Neuropathy in mice is Age-Dependent

**Fig. 6:**
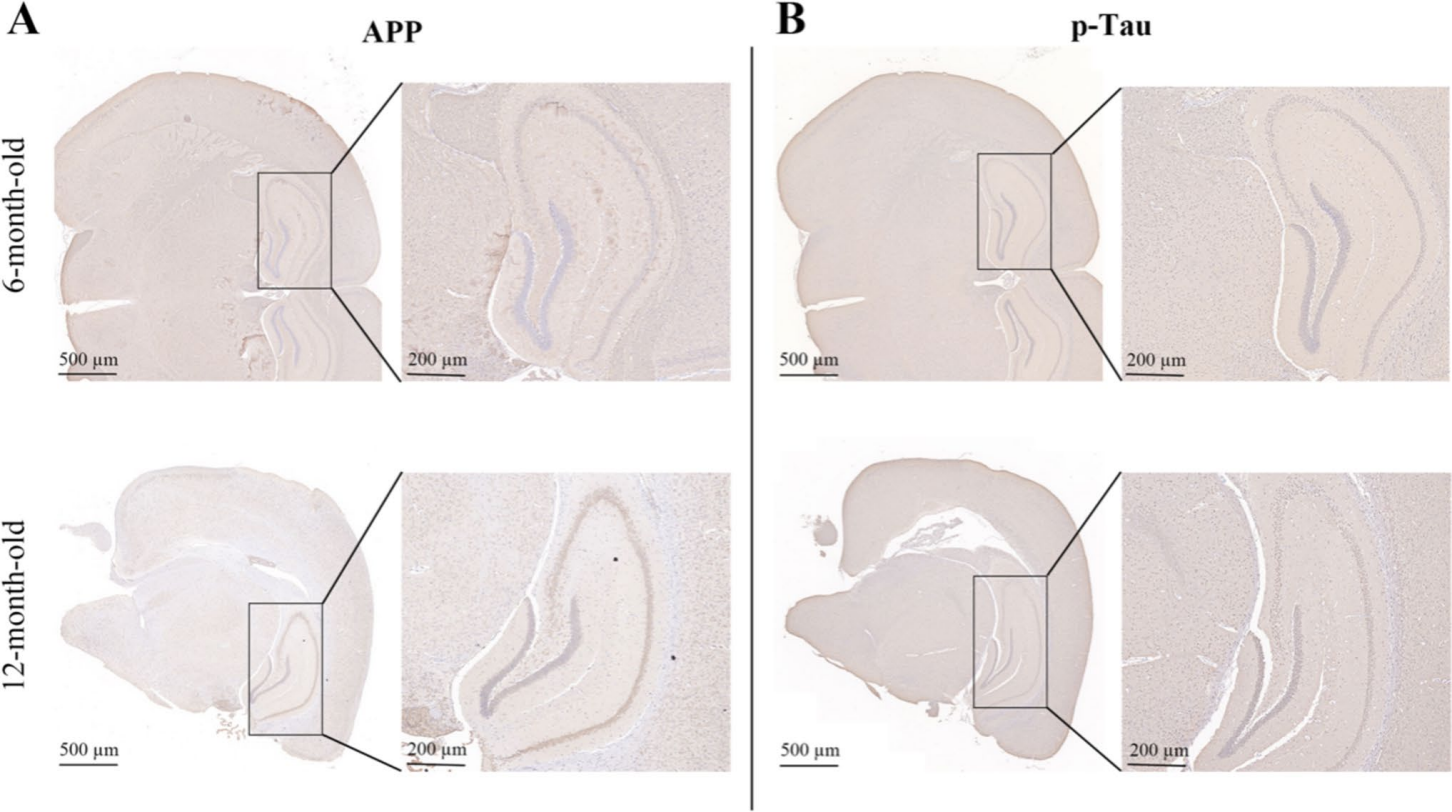
AD-like disease induced by IE2 increases with age in IE2 transgenic mice. **A** Representative IHC from 6-month-old and 12-month-old IE2 mice showing that APP was upregulated with age. The inset shows the 5 $\times$ magnification of the corresponding panel. Scale bar, 1 mm (panels); 50 µm (insets). **B** Representative IHC from 6-month-old and 12-month-old IE2 mice showing that p-Tau was upregulated with age. The inset shows the 5 $\times$ magnification of the corresponding panel. Scale bar, 1 mm (panels); 50 µm (insets)

To explore the effect of IE2 on mice of different ages, 6-month-old and 12-month-old IE2 transgenic mice were used to detect the expression of Aβ and p-Tau, and wild-type littermates were used as a control. Immunohistochemical staining showed that the expression of Aβ and p-Tau were significantly higher in IE2 mice compared to controls, especially in the 12-month-old IE2 mice (Fig. 6). To further verify our results, we also used lentivirus-infected mice for detection. The expression of Aβ and p-Tau in 12-month-old LV-IE2 mice was significantly higher than that in 6-month-old mice (Fig. S3).This suggested that the neuropathy caused by IE2 is age-dependent.

## Discussion

Prior studies have shown that HCMV infection is lifelong, with the virus maintained in a state of latency or low-level persistence in healthy individuals [5]. It can reactivate infection in immunocompromised populations, especially the elderly. HCMV is known to infect the central nervous system (CNS), and the proinflammatory response of the CNS to HCMV infection may be a factor that leads to chronic neurodegenerative diseases such as Alzheimer’s disease [29, 30]. Researchers have evaluated the correlation between these immune reactions and the pathological markers of AD in CMV-seropositive subjects. The CMV-specific serum IgG antibody level has been found to be significantly correlated with NFT formation, and CMV infection has been found to markedly induce the formation of Aβ plaques[31]. IE2, immediately-early protein of HCMV, has been shown to affect brain development and cognition in mice, which manifests as synapse loss in the hippocampus and cerebral cortex, leading to learning and cognitive impairment [9].

In the current study, we used IE2 transgenic mice and single-cell sequencing to explore the effects of HCMV IE2 in neurodegenerative disease in mice. Subsequently, DEGs in the microglia of IE2 transgenic mice were identified, including upregulated genes (ApoE, ISG15, CD83) and downregulated genes (Tmem119, P2ry12), which were associated with disease and homeostasis, respectively. We uncovered a gene expression signature similar to DAM in a subset of microglia isolated from the brain of IE2 transgenic mice and started to untangle the controversy regarding the beneficial or detrimental functional roles of these cells. IE2 transgenic mice were found to be rich in DAM risk factors, indicating the potential role of these proteins in disease pathogenesis.

The results indicated that IE2 might be an important factor driving microglial type changes under normal and pathophysiological conditions. By examining the cellular pathways correlated with IE2 in microglia, we identified novel associations between IE2 expression and immune response pathways, neurodegenerative disease, and cellular processes. IE2, as an immediate early protein, is crucial to inducing the HCMV cleavage gene cascade. A long-term viral infection state is simulated in IE2 transgenic mice. Therefore, we speculated that there is a relationship between the neurodegenerative disease caused by IE2 and the immune system. This finding suggests that the pathological upregulation of IE2 may play a crucial role in the nervous system. Previous experiments have demonstrated that overexpression of the IE2 gene can lead to loss of synapses, neurons, and hippocampal volume [10].

In recent studies, researchers found that AD is a chronic autoimmune disorder, wherein necrotic (not apoptotic) breakdown products trigger a significant release of Aβ [32–34]. The subsequent breakdown products of necrotic neurons elicit further release of Aβ, leading to a chronic, self-perpetuating cycle [34]. According to the analysis of the single-cell data, the expression of IE2 is accompanied by the activation of microglia, and this phenomenon is typically thought to contribute to brain inflammation[35, 36]. Indeed, activated microglia perform the function of antigen-presenting cells in autoimmune diseases of CNS [37, 38].

DEG analysis showed that the activated microglia express MHC II molecules, which enable antigen presentation to CD4^+^ T cells. It has been reported that MHC II is a marker of microglial activation and that AD patients have higher level of MHC II positive microglia than control subjects [39, 40]. Matsushima et al. demonstrated that MHC II molecules aggravate the neurodegenerative symptoms and neuropathology of globoid leukodystrophy in mice [41]. Our immunochemical results showed that Aβ plaques and the expression of p-Tau were markedly increased in 12-month-old IE2 transgenic mice compared to control mice (Figs. 5 and 6). The single-cell sequencing data provided us with partial evidence of the correlation between HCMV and neurodegenerative disease, which explained the learning and memory impairment observed in IE2 transgenic mice in previous studies [9, 42]. Taken together, this evidence shows that microglia may also function as an element of the adaptive immune system with the IE2 expression.

Due to the specificity of the natural host of HCMV, it is difficult to obtain HCMV-infected human samples. Although IE2 transgenic mice simulate the expression process of viral proteins after HCMV infection in vivo to a certain extent, there are still some limitations. Despite these limitations, our study opens several avenues of investigation: (1) based on our mouse model, it is now possible to explore the response of microglia to IE2 expression; (2) we identified at least one subset of microglia that may be associated with AD-like disease, and this should be prioritized for further validation; (3) relevant evidence for HCMV and neurodegenerative disease was presented.

**Fig. 7:**
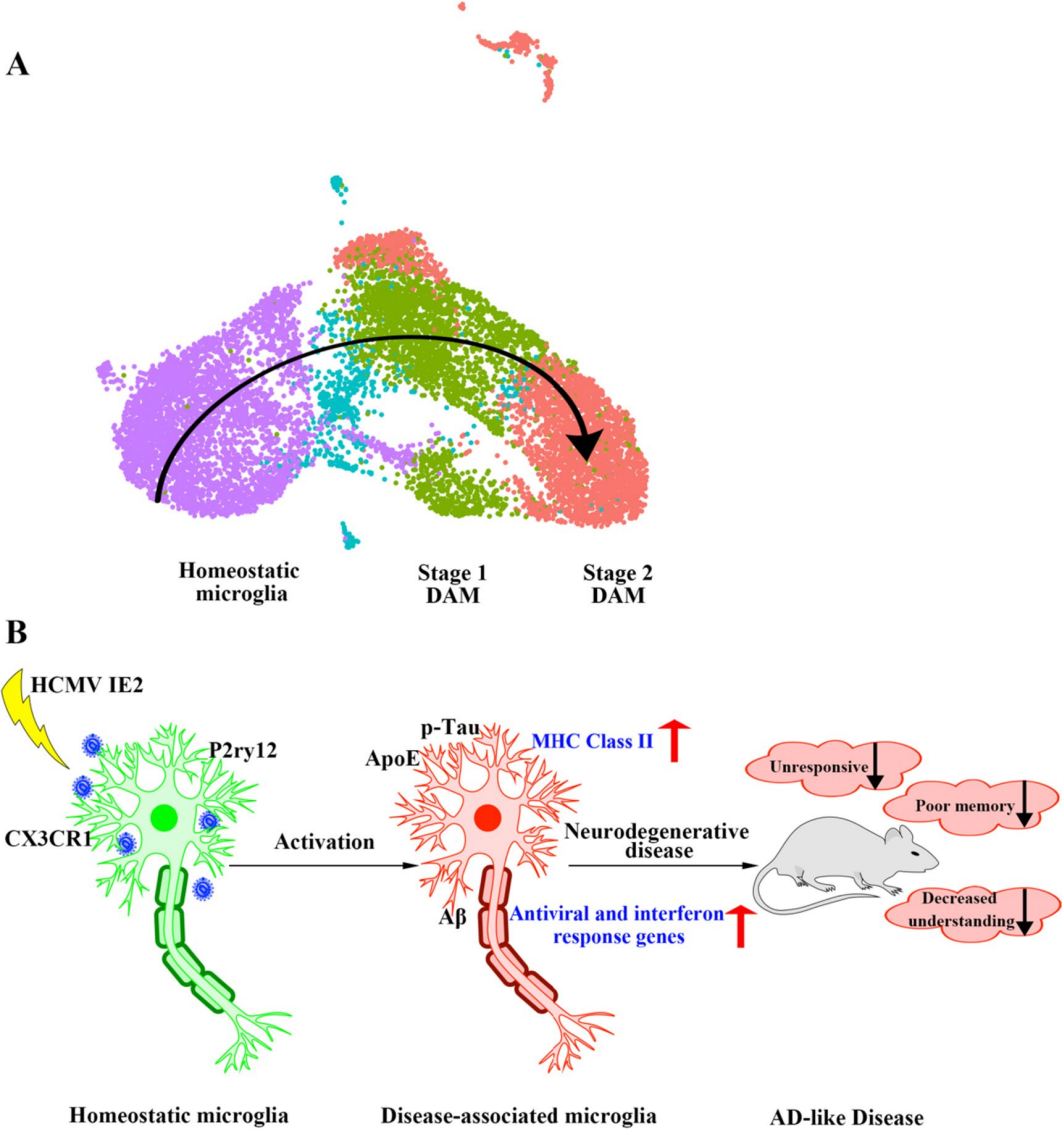
AD-like disease is regulated by microglia activation mechanism induced by HCMV IE2. **A** Schematic illustration showing microglia switching from homeostatic to stage 1 (an intermediate state) and stage 2 DAM following signals such as those associated with AD pathology and aging. **B** Features of mouse neurodegenerative disease caused by microglia activation induced by IE2. Key genes are shown below each condition. Arrows indicate up (red) or down (black) regulation in the specific stage

The successful construction of IE2 transgenic mice in this study provides a new model for understanding HCMV infection in vitro. By analyzing mice expressing IE2 in the hippocampus, we proposed a hypothesis that HCMV infection causes the activation of microglia, which leads to a series of immune responses in the brain, and then promotes the occurrence and development of AD-like neurodegeneration (Fig. 7). Taken together, our results are essential for a more comprehensive understanding of microglial activation caused by HCMV infection and the series of pathological reactions that occur after microglial activation. The findings of this study may provide new ideas for the early detection of AD-related neurodegenerative disease and the development of targeted microglial therapy.

### Supplementary Information

ESM 1 (XLS 61.4 kb)

## Data Availability

Data and all materials except for HCMV IE2 transgenic mice are available on request from the corresponding author. HCMV IE2 transgenic mice are not commercially available. For availability and use of HCMV IE2 transgenic mice, contact their developer Dr. Wang.
